# Infection success of *Echinoparyphium aconiatum* (Trematoda) in its snail host under high temperature: role of host resistance

**DOI:** 10.1186/1756-3305-7-192

**Published:** 2014-04-21

**Authors:** Katja Leicht, Otto Seppälä

**Affiliations:** 1 Eawag, Swiss Federal Institute of Aquatic Science and Technology, Dübendorf 8600, Switzerland; 2 Department of Biological and Environmental Science, University of Jyväskylä, Seminaarinkatu 15, Jyväskylä 40014, Finland; 3 ETH Zürich, Institute of Integrative Biology (IBZ), Zürich 8092, Switzerland

**Keywords:** *Echinoparyphium aconiatum*, Global climate change, Heat wave, *Lymnaea stagnalis*, Resistance to infection, Host-parasite interaction, Experimental assessment

## Abstract

**Background:**

Extreme weather events such as summer heat waves become more frequent owing to global climate change and are predicted to alter disease dynamics. This is because high temperatures can reduce host immune function. Predicting the impact of climate change on host-parasite interactions is, however, difficult as temperature may also affect parasite infective stages and other host characteristics determining the outcome of interaction.

**Methods:**

Two experiments were conducted to investigate these phenomena in a *Lymnaea stagnalis–Echinoparyphium aconiatum* (Trematoda) interaction. In the first experiment, the effects of exposure of snails to experimental heat waves [maintenance at 25°C vs. 15°C (control)] with different durations (3 days, 7 days) on the infection success of parasite cercariae was examined. In the second experiment, the infection success was examined under similar conditions, while controlling for the possible temperature effects on cercariae and at least partly also for host physiological changes that take place rapidly compared to alterations in immune function (exposure to cercariae at intermediate 20°C).

**Results:**

In the first experiment, increased infection success at 25°C was found independently of the duration of the heat wave. In the second experiment, increased infection success was found only in snails maintained at 25°C for 7 days, a treatment in which snail immune defence is known to be impaired.

**Conclusions:**

These results suggest that the effects of host resistance in determining overall parasite infection success can be overridden by effects of temperature on parasite transmission stages and/or alterations in other host traits than immune defence.

## Background

The outcomes of host-parasite interactions and therefore disease dynamics are often affected by environmental conditions (see [1,2] for review). Especially as ambient temperature can heavily influence these dynamics [3,4]. Owing to anthropogenic climate change, understanding such effects is increasingly important as climate change leads to an increase in average air temperature and more frequent occurrence of extreme weather events such as summer heat waves [5,6]. The effect of high temperature on host-parasite interactions is often assumed to take place via reduced resistance of hosts to infections due to impaired immune function [7,8]. This is because immune defence is typically considered to be the main physiological barrier against parasites (see [9] for review).

Predicting the effects of temperature on host-parasite interactions based only on the knowledge on host immune defence can, however, be difficult. This is because parasite infection strategies as well as other host characteristics than immune function can be important in determining the outcome of interactions. For instance, temperature can directly affect parasite infective stages (e.g. survival, mobility) thus modifying their infection success [10-13]. Similarly, in ectothermic species, temperature affects host metabolism and could modify their physiological traits (see [14] for review). For example, metabolic products, excreted into the environment by the organisms are used for host finding by some parasite species (see for review [15-17]), and temperature could alter excretion of such chemical host cues. However, the role of the above effects is likely to be system specific [18-20]. This could cause high variation in the effects of temperature across different host-parasite interactions complicating the predictions of the effects of climate change [21]. Hence, examining the infection success of parasites under high temperature and the role of altered host defences in determining this is in high demand.

This study focused on the effect of high ambient temperature on the infection success of *Echinoparyphium aconiatum* (syn. *Pseudoechinoparyphium echinatum*; Trematoda) cercariae in its snail host *Lymnaea stagnalis*. In this system, high temperature is known to reduce several immune defence traits of snails [22,23], and thus snails can be assumed to become less resistant to infections during heat waves. This effect, however, is seen only when snails are maintained at high temperature for one week or longer [23]. The parasite *Echinoparyphium aconiatum* is one of the most common trematode species infecting *L. stagnalis*[24-26]. It has a complex life-cycle including snails as first and second intermediate hosts, and snail-eating birds as a definitive host [27]. Cercariae released from the first intermediate host use chemical cues (e.g. amino acids) to find their second intermediate host from the environment [15,18,28], which they enter through the urinary orifice and develop into encysted metacercariae mostly in the hepatopancreas.

Here, the effects of experimental heat waves on the interaction between *L. stagnalis* and *E. aconiatum* cercariae were examined in two experiments. The first experiment investigated their effects on the overall parasite infection success. The second experiment, examined infection success by controlling for the possible temperature effects on cercariae and at least partly for host physiological changes that take place rapidly compared to alterations in immune function which are slow in this system [23]. As experimental temperatures of 15°C [a common temperature in habitats of snails (A. Laurila, 2010, unpublished data; U. Tobler, 2010, unpublished data)] and 25°C [a temperature that occurs intermittently in ponds during hot summers (A. Laurila, 2010, unpublished data; U. Tobler, 2010, unpublished data)] were used. As only long-term (one week or longer) maintenance at high temperature is known to impair immune function of snails [23] the effects of both short- (3 days) and long-term (7 days) heat waves were tested. We predict overall parasite infection success to increase (experiment 1) and host resistance to infection to decrease (experiment 2) after long-term maintenance of snails at high temperatures. If temperature affects parasite transmission stages and/or other host traits than immune defence we expect host resistance to be a poor predictor for parasite infection success.

## Methods

### Experimental animals

Experimental snails came from a laboratory stock population (F_2_ generation) originating from a pond in Zürich, Switzerland (47°22′N, 8°34′E). The population was maintained in water tanks (temperature ranging from 12°C to 20°C, but being close to 15°C for most of the year) for one year before the experiment. Five days prior to the experiment (see below), experimental snails (shell length: 27.6 - 42.6 mm) were randomly chosen from the population and placed individually in plastic cups filled with 200 ml of aged tap water at 15°C to acclimatize them to the experimental conditions. Snails were fed with spinach *ad libitum*, and water in the cups was changed every second day during this period and throughout the following experiments.

Snails releasing *E. aconiatum* cercariae for the experiment originated from ponds in Biengarten, Germany (49°39′N, 10°49′E). With using an allopatric parasite a coevolutionary history between the host and the parasite could be avoided. Snails collected from the field were brought to the laboratory and placed individually in plastic cups filled with 40 ml of water. Snails infected with *E. aconiatum* were identified by observing the morphology of released cercariae (see [25]). Infected snails were maintained in boxes, with 10 to 15 snails in each, filled with 6 l of aged tap water at 20°C (a typical temperature at the collection site at the time of sampling; own obs.) and fed with spinach *ad libitum* between collecting and use in the experiment.

### Experimental design

The study was conducted in two experiments that ran simultaneously. The first experiment examined the overall effect of high temperature on the infection success of *E. aconiatum* in *L. stagnalis*. Experimental snails were maintained as described above and randomly assigned into one of the two temperature (15°C, 25°C) and maintenance time (3 days, 7 days) treatments (20 snails per treatment combination). Water in the cups was changed once more after the maintenance period and snails were exposed to parasite cercariae (see below) at their respective maintenance temperatures.

The second experiment examined the effect of high temperature on the resistance of snails to infection when controlling for the potential direct effects of temperature on parasite cercariae and partly also for host physiological changes that take place rapidly compared to alterations in immune function and could affect the outcome of interaction (see the Discussion for potential mechanisms). Experimental snails were maintained as in experiment 1. After the maintenance period, snails were removed from their cups and transferred into new cups filled with aged tap water at 20°C (temperature at which the snails producing cercariae were maintained). Immediately after the transfer, snails were exposed to *E. aconiatum* cercariae (see below).

After the maintenance period in experimental treatments (see above), snails were exposed to freshly emerged (5–30 min old) parasite cercariae. To gain cercariae, a total of 60 infected snails were transferred into cups filled with fresh water (i.e. no previously released cercariae) and emerged cercariae were collected under a microscope. In the parasite exposures, four to five different host snails were used to gain a total of 20 cercariae that were introduced into the cup of each experimental snail. Which experimental snail received parasites from which infected snails was determined by chance. Thus, each infected snail contributed equally to the mix of cercariae used for infections, and the genetic (i.e. clonal) composition of parasites varied among exposed snail individuals. The exposure dose of 20 cercariae per snail was used as it allows examining infection success as a quantitative trait [29,30], and as parasite intensities in the wild commonly vary between 10 and 30 metacercariae per snail (own obs.). Snails were exposed to the parasites for 24 h. This exposure time is sufficient for the parasites to invade the snails and form metacercariae as they are only infective for approximately 24 h after emergence [31]. After that, snails were removed from the cups and their shell length was measured to the nearest 0.1 mm. Then, snails were removed from their shells and parasites that successfully infected the snails (i.e. encysted as metacercariae into snail tissues) were counted under a microscope.

### Statistical analyses

Variation in parasite infection success was analysed using generalized linear models for both experiments. In these models, the proportion of parasites successfully infecting the snails (*x*/20) was used as a binomial response variable (logit link function), and maintenance temperature (15°C, 25°C) and maintenance time (3 days, 7 days) were used as fixed factors. Because of a significant interaction between the factors in the second experiment (see Results), the data were further analysed separately for different maintenance times to estimate the effect of maintenance temperature in detail. In these analyses, generalized linear models as above with maintenance temperature as a fixed factor were used. Size of snails is known to partly determine the infection success of *E. aconiatum*[29]. In this study, however, the shell length of the snails did not differ among treatment groups at the end of the experiment (Additional file 1). Furthermore, using shell length as a covariate in the above models led to significant interactions between factors and the covariate. Therefore, shell length was not included into the final models. All statistical analyses were performed using IBM SPSS Statistics Version 19.0 software (Armonk, NY: IBM Corp.).

## Results

**Figure 1:**
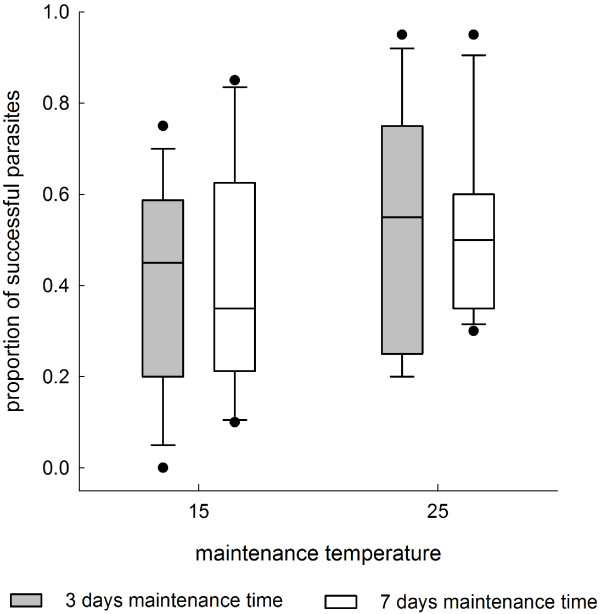
**Infection success of *E. aconiatum *cercariae in the first experiment.** Proportion of encysted metacercariae in snails maintained at different temperature treatments for three or seven days before parasite exposure (20 cercariae per snail, 11 to 20 snails per treatment group) when the snails were exposed to parasite cercariae at their respective maintenance temperature. Data are shown as box plot with median, 25th (lower box) and 75th (upper box) quartiles; whiskers represent the values within the 1.5 interquartile range (outliers are shown as ●).

In the first experiment, the proportion of cercariae that successfully infected the snails was higher in individuals maintained and exposed to parasites at 25°C than in snails maintained and exposed at 15°C (Table 1, Figure 1). This effect was independent of maintenance time (Table 1, Figure 1).

**Table 1:** **GLM for the proportion of *E. aconiatum *** **cercariae infecting *L. stagnalis *** **in the first experiment**

	**Wald chi-square**	**df**	***p***
Temperature (T)	16.921	1	<0.001
Maintenance time (D)	0.223	1	0.637
T × D	0.715	1	0.398

**Figure 2:**
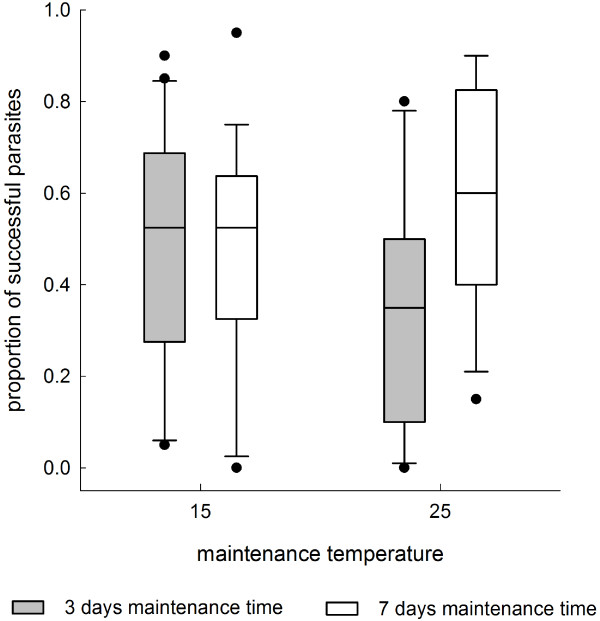
**Infection success of *E. aconiatum *cercariae in the second experiment.** Proportion of encysted metacercariae in snails maintained at different temperature treatments for three or seven days before parasite exposure (20 cercariae per snail, 11 to 20 snails per treatment group) when the snails were exposed to parasite cercariae at 20°C. Data are shown as box plot with median, 25th (lower box) and 75th (upper box) quartiles; whiskers represent the values within the 1.5 interquartile range (outliers are shown as ●).

In the second experiment where the snails were exposed to parasites at 20°C, maintenance temperature of snails before parasite exposure did not have a significant main effect on parasite infection success (Table 2, Figure 2). Its effect, however, depended on maintenance time indicated by a significant interaction between the factors (Table 2). Therefore, the effect of maintenance temperature was analysed separately for different maintenance time treatments. In the long-term treatment, higher parasite infection success was found in snails maintained at 25°C than at 15°C (generalized linear model: Wald Chi-Square = 6.736, *p* = 0.009, Figure 2). In the short-term treatment, maintenance at 25°C before parasite exposure led to fewer infections than maintenance at 15°C (generalized linear model: Wald Chi-Square = 11.182, *p* = 0.001, Figure 2).

**Table 2:** **GLM for the proportion of *E. aconiatum *** **cercariae infecting *L. stagnalis *** **in the second experiment**

	**Wald chi-square**	**df**	***p***
Maintenance temperature (T)	0.466	1	0.495
Maintenance time (D)	16.379	1	<0.001
T × D	17.778	1	<0.001

## Discussion

In this paper, we show that exposure of *L. stagnalis* snails to high temperature increased the infection success of *E. aconiatum* cercariae. This, however, was not only due to decreased host resistance. In the first experiment where the snails were exposed to cercariae at their respective maintenance temperature, the proportion of parasites that successfully infected the snails was higher at 25°C compared to 15°C both in short- and long-term treatments. This indicates that other factors than the altered immune function (see [22,23]) are important in determining infection success as immune function of *L. stagnalis* is known to be reduced only after seven days of exposure to high temperature (see [23]). In the second experiment where the resistance of snails against the infection was examined by controlling for the possible direct effects of temperature on parasite infective stages and partly for temperature-dependent changes in host metabolic and physiologic rate (i.e. exposure at 20°C), infection success was higher only when the snails were maintained at 25°C for seven days before parasite exposure. This indicates that the resistance of snails against *E. aconiatum* is affected by temperature, and confirms that other factors need to override its effect in determining the infection success of the parasite.

The observed effect of temperature on overall parasite infection success could take place via at least two different mechanisms that may override the effect of altered host resistance. First, temperature could directly affect parasite cercariae by altering their mobility, survival, and infectivity [10,13,31,32]. For instance, an effect on the activity of cercariae can alter the contact probability between the parasite and the host leading to changed transmission efficiency [10,13,32]. Similarly, altered efficiency in the use of energy reserves can change infectivity of cercariae [10,12]. Thus, an increase in the performance of parasites at high temperature may enable cercariae to overcome host defences more easily. Second, ambient temperature may also affect other host traits than immune function, which could alter the outcome of the interaction. For instance, echinostome cercariae are able to find snails from the environment using chemo-orientation by responding to the micromolecules excreted by the snails [15,18,28]. These responses depend on the total amount of such host cues and also on their chemical composition [15,18,28]. Thus, environmental factors that alter the metabolism and physiology of snails could modify the excretion of such molecules altering exposure to parasite transmission stages. For example, physiological effects of food processing are known to predispose snails to *E. aconiatum* cercariae [29]. Similarly, ambient temperature could change the amount and composition of such chemical cues in the environment by altering snail metabolism. The relative importance of changes in host cues in the environment compared to direct temperature effects on parasite larvae might be large as chemo-orientation allows direct movement towards the host [18]. However, the actual role of these potential mechanisms remains to be investigated.

Interestingly, in the first experiment, the combined effect of reduced resistance due to long-term maintenance at high temperature and possible direct effects of temperature on parasite transmission stages and/or differences in host physiology apart from immune defence did not lead to higher infection success compared to short-term exposures. It is possible that the maximum infection success of cercariae was already reached in both treatments as not all parasites may be able to infect the snails (e.g. due to intrinsic factors of the parasite such as mortality or infectivity). The highest observed infection success of approximately 60% is lower than what has been reported for this species earlier [29,33]. The use of an allopatric host may have reduced the maximum infection success in this study (see [34] for review, [35]). Moreover, in the second experiment, the proportion of encysted parasites when the snails were maintained at high temperature for three days before parasite exposure was lower than when the snails were maintained at 15°C. This was unexpected as in our earlier study [23], none of the examined immune parameters (i.e. haemocyte concentration, phenoloxidase-like activity, and antibacterial activity of snail haemolymph) was altered by short-term maintenance at high temperature. It is important to note, however, that immune responses of snails to *E. aconiatum* are currently not well understood. Thus, it could be that some other immune traits that were not examined in our earlier study [23] determine the resistance of snails to this parasite species. Such parameters could respond differently to high temperature compared to the ones examined earlier [8], which could alter snail resistance already after short-term maintenance at high temperature. Potential candidates for such mechanisms are, for example, reactive oxygen species such as hydrogen peroxide [36] and lectins such as fibrinogen-related proteins (see [37] for review), which have been reported to be important in snail defences against trematodes in other species.

## Conclusions

Exposure of snails to high temperature increased the infection success of *E. aconiatum* cercariae independently of the length of the experimental heat wave snails had experienced. However, when controlling for the effects of temperature on parasite transmission stages and potential temperature-dependent changes in host metabolism and physiology that may take place rapidly compared to changes in immune defence (e.g. excretion of chemical cues), only long-term maintenance at high temperature led to increased infection success. This suggests that such factors overrode the effects of temperature on host resistance in determining the infection success under high temperature. Our results suggest that heat waves can lead to higher parasite infection success and thus increase the probability of epidemics. However, environmental conditions can also influence at other stages of parasite life cycles. For example, the production and release of cercariae in the snail hosts is known to depend on environmental conditions in several parasite species [38-40]. Additionally, environmental stress can cause high mortality among infected hosts [41-43]. Since parasite population dynamics are determined by a balance between parasites within-host growth and reproduction, host and parasite mortality, and parasite transmission rate [44,45], all of which may vary across host and parasite species, more studies focusing on different steps of parasite life cycles are needed to assess the overall effect of climate change on disease dynamics.

## Competing interests

The authors declare that they have no competing interests.

## Authors’ contributions

KL and OS designed and implemented the experiments, and performed the statistical analyses. KL wrote the manuscript. OS revised the manuscript. Both authors read and approved the final manuscript.

## Supplementary Material

Additional file 1 Analysis of differences in the shell length of snails among treatments at the end of the experiments. Click here for file
