# Supplementary material for: Infection success of Echinoparyphium aconiatum (Trematoda) in its snail host under high temperature: role of host resistance
Source: Parasit Vectors. 2014 Apr 21;7:192. doi: 10.1186/1756-3305-7-192 (PMC4021695; doi:10.1186/1756-3305-7-192)
Supplement: Additional file 1 — Analysis of differences in the shell length of snails among treatments at the end of the experiments. [file 1756-3305-7-192-S1.pdf]

Additional file 1

Statistical analyses

To analyse differences in the shell length of snails among treatments at the end of the experiments, generalized linear models were performed for both experiments. In these models, shell length was used as a response variable (identity link function), maintenance temperature (15°C, 25°C) and maintenance time (3 days, 7 days) before parasite exposure were used as fixed factors.

Results

Shell length at the end of the experiments did not depend on experimental treatments (Tables A1, A2).

Table A1: GLM for the shell length of *L. stagnalis* by maintenance temperature (15°C, 25°C) and maintenance time (3 days, 7 days) before parasite exposure in the first experiment.

	Wald Chi-Square	df	*p*
maintenance temperature (T)	0.201	1	0.654
maintenance time (D)	3.210	1	0.073
T × D	0.136	1	0.712

Table A2: GLM for the shell length of *L. stagnalis* by maintenance temperature (15°C, 25°C) and maintenance time (3 days, 7 days) before parasite exposure in the second experiment.

	Wald Chi-Square	df	*p*
maintenance temperature (T)	1.408	1	0.235
maintenance time (D)	0.762	1	0.383
T × D	0.010	1	0.921
